# Impact of the Age of Cecal Material Transfer Donors on Alzheimer’s Disease Pathology in 5xFAD Mice

**DOI:** 10.3390/microorganisms9122548

**Published:** 2021-12-09

**Authors:** Francesco Valeri, Malena dos Santos Guilherme, Fuqian He, Nicolai M. Stoye, Andreas Schwiertz, Kristina Endres

**Affiliations:** 1 Department of Psychiatry and Psychotherapy, University Medical Center, Johannes Gutenberg University Mainz, 55131 Mainz, Germany; francescovaleri47@gmail.com (F.V.); malena-guilherme@gmx.de (M.d.S.G.); nicolai.stoye@gmx.net (N.M.S.); 2 The Center of Gerontology and Geriatrics, West China Hospital of Sichuan University, Chengdu 610041, China; lotusmart_2007@126.com; 3 MVZ Institute fuer Mikrooekologie GmbH, 35745 Herborn, Germany; Andreas.Schwiertz@mikrooek.de

**Keywords:** aging, amyloid, antibiotics, dysbiosis, fecal material transplant, microbiome

## Abstract

Alzheimer’s disease is a progressive neurodegenerative disorder affecting around 30 million patients worldwide. The predominant sporadic variant remains enigmatic as the underlying cause has still not been identified. Since efficient therapeutic treatments are still lacking, the microbiome and its manipulation have been considered as a new, innovative approach. 5xFAD Alzheimer’s disease model mice were subjected to one-time fecal material transfer after antibiotics-treatment using two types of inoculation: material derived from the caecum of age-matched (young) wild type mice or from middle aged, 1 year old (old) wild type mice. Mice were profiled after transfer for physiological parameters, microbiome, behavioral tasks, and amyloid deposition. A single time transfer of cecal material from the older donor group established an aged phenotype in the recipient animals as indicated by elevated cultivatable fecal Enterobacteriaceae and Lactobacillaceae representative bacteria, a decreased Firmicutes amount as assessed by qPCR, and by increased levels of serum LPS binding protein. While behavioral deficits were not accelerated, single brain regions (prefrontal cortex and dentate gyrus) showed higher plaque load after transfer of material from older animals. We could demonstrate that the age of the donor of cecal material might affect early pathological hallmarks of Alzheimer’s disease. This could be relevant when considering new microbiome-based therapies for this devastating disorder.

## 1. Introduction

In recent years, neuroscientists have become increasingly interested in the so-called gut–brain-axis (as reviewed in: [1,2,3]). Since the origin of the sporadic form of AD is still enigmatic, it is highly attractive to explore possible novel pathogenicities and their impact on the disease to unravel underlying mechanisms and thereby identify new urgently needed therapeutic treatment options. For an estimated number of 30 million patients suffering from the devastating symptoms of AD that include memory loss but also severe changes in personality traits (e.g., [4]), it is sad to say that still only symptomatic treatments are available, e.g., acetylcholine esterase inhibitors (for a systematic review and meta-analysis see [5]). A growing number of investigations reported the presence of an altered gut or stool microbiome in AD patients [6,7] and rodent disease models [8,9]. For example, the genus *Ruminococcus* has been identified to be reduced, while e.g., *Blautia* and *Odoribacter* increased in patients or disease models (reviewed in [10]). This may indicate an AD-associated microbiome and allots the dysbiosis of microbial commensals an important role in pathogenesis. However, these observations might also only describe a side effect ancillary to the pathogenic mechanisms. It has long been described that AD patients have an early reduction of body weight before clinical symptoms occur [11,12,13] and our own study using the 5xFAD mouse model revealed a transient reduction in tryptic activity within the gut, accompanied with an altered microbiome as compared to wild type littermates [9]. This, together with the reported changes in patients’ eating habits and eating ability [14,15], might be sufficient to explain an alteration of microbial composition in AD. Until these observational associations can be mechanistically verified, any therapeutic approaches targeting the microbiome must be conducted with caution.

Transfer of microbiome-containing material (designated as fecal material transfer; FMT) came into focus as a treatment option in several recent reviews (for example [16]). In intestinal diseases, such as recurrent *Clostridium difficile* infection, this is an already applied but still debated therapy [17,18]. For neurological disorders, data are still scarce. A recent review investigated 34 studies with FMT from healthy donors [16]: for autism spectrum disorders, epilepsy, multiple sclerosis, and Parkinson’s disease animal model studies but also single clinical studies with human patients were included. For neuropathic pain, stroke, and Guillain-Barré syndrome, only few animal studies have been published so far. The outcome of these studies showed mainly positive effects of FMT. For multiple sclerosis, decreased disease severity and increased walking ability was observed (e.g., [19,20]); for epilepsy, increased seizure threshold in the animal study and decreased seizure frequency in patients were observed [21,22]. For AD no such investigation on transfer of healthy donor material into human patients is available and only a few studies on the disease aggravating effect of the transfer of fecal material from AD mice into germ-free or antibiotics-treated mouse models have been published [23,24]. One study from 2019 reported on the transfer of wild type gut microbiome into AD model mice: Sun and colleagues [25] used the APPswe/PS1dE9 transgenic (Tg) mouse model to investigate putative neuroprotective effects of FMT from healthy donors. Mice were administered with 0.2 mL fresh fecal solution from wild type mice intragastrically once daily for four weeks after pre-treatment with antibiotics for three days. Subsequently, performance in the Morris water maze test increased, Aβ peptide levels decreased as well as phospho-Tau amount and levels of synapsin and PSD-95 were restored. In this study, recipient as well as donor mice were of six months of age.

Aging is the main risk factor for developing sporadic AD (e.g., [26]). Many of the pathological changes found in AD are comparable to cognitively healthy aging, except for severity. With age, there is a reduction in brain volume, enlargement of the ventricles, and loss of synapses, dendrites and connectivity within functional networks in certain cortical regions [27,28]. Therefore, AD could be described as an accelerated form of aging. In 2020, 3% of people, aged 65–74, 17% of people aged 75–84, and 32% of people aged 85 or older were diagnosed with AD [29]. According to this, the prevalence of AD increases exponentially with age, doubling approximately every 5 years after age 65 [30]. Due to the high relevance of age for AD pathogenesis, this study aimed to elucidate the impact of age of the donor for FMT in AD. To address this question, we inoculated antibiotics-pre-treated 5xFAD model mice via gavage with either cecal suspension from age-matched wild type animals or with material from mice at mid-age (one year old). Subsequently, we analyzed microbial composition, behavior, and pathological hallmarks of these animals post treatment.

## 2. Materials and Methods

### 2.1. Animals

B6SJLTg (APPSwFlLon, PSEN1-M146L-L286V)6799Vas/Mmjax (5xFAD, RRID:IMSR_JAX:032884 [31]) mice (Jackson Lab, Bar Harbor, ME, USA) were maintained by crossbreeding with C57BL/6J background as described in [32]. All animals were reared in a 12 h light/dark cycle with food (Ssniff Spezialdiäten GmbH, Soest, Germany) and water available *ad libitum*. All procedures were performed in accordance with the European Communities Council Directive regarding care and use of animals for experimental procedures and were approved by local authorities (Landesuntersuchungsamt Rheinland-Pfalz; approval number G 17-1-035). The number of animals that entered the single experimental procedures was calculated taking into account outcome of former behavioral experiments (e.g., pharmacological interventions). Heterozygous male mice were mated with female wild type mice. After one week, the male was removed; the female was put on antibiotics-supplemented drinking water after reaching the third week of pregnancy with the following components: Gentamicin (0.083 mg/mL), Vancomycin (0.042 mg/mL), Metronidazole (0.167 mg/mL), Neomycin (0.042 mg/mL), Ampicillin (0.083 mg/mL), Colistin (500 U/mL), and Cefaperazone (0.083 mg/mL) (all Sigma Aldrich, Steinheim, Germany or Cayman Europe, Tallinn, Estonia). This treatment was based on [33] but Kanamycin was omitted due to its teratogenic property [34]. Concentrations were additionally reduced to 2/3 of the initially described dosages to spare health of the dams and adapted to the mean daily drinking volume of a mouse (4 mL). Dams were inspected on a regular base for pup-care and self-maintenance and showed no signs of altered behavior. This was also reflected by litter sex-composition and an even increased mean pup number per litter (see Supplementary Table S1). When pups were born, antibiotics dosage was reduced to 1/50 and animals kept on autoclaved food. With three weeks, mice were weaned, genotyped and kept overnight on autoclaved tap water to wash out enteral antibiotics before transfer of the cecal material. This step was conducted to ascertain colonization by FMT on the following day. Subsequently, animals received drinking water without antibiotics.

Only transgenic offspring without any sign of health impact (ragged fur, low body weight) was used for receiving FMT at an age of four weeks. Both sexes were included, and animals randomly assigned to the groups “young” and “old” (alternating assignment following the order of the ear numbers), meaning assigned to treatment with cecal material derived from young or old donors. Wild type mice served as control for the behavioral investigations. These animals were not treated with antibiotics but received a single mock-gavage with PBS to mimic the FMT procedure and the therewith-experienced stress, which might affect the outcome of behavioral tasks. This mock-gavage was also conducted for transgenic animals without FMT for comparison of bacterial viable representatives with those of FMT receivers. All animals were single-caged after the FMT or mock-administration in fresh cages to prevent transfer of gut microbiota by coprophagy. The single animal was accounted as the experimental unit.

### 2.2. Cecal Material Transfer

Wild type animals aged 4 weeks (young donors) or aged one year (old donors) were sacrificed by decapitation after isoflurane anesthesia and content of caecum collected in argon-flushed PBS with glycerin (1:10 *w*/*v* in 50% glycerol/PBS following [35]). Aliquots were shock-frozen with liquid nitrogen and stored at −80 °C for maximally 1 month. To equalize for individual differences, two animals, one male and one female, were used per preparation of the to-be-transferred material. At the day of FMT the suspension was further diluted 1:5 prior to oral gavage of 150 µL per mouse. In accordance with previous publications [36,37,38], we designate the transfer of caecum suspensions here in the following as FMT.

### 2.3. Quantitation of Bacterial CFU

Lactobacillaceae and Enterobacteriaceae as representative families of gut commensals were assessed by counting colony forming units (CFUs) as described previously [39]. In brief, freshly, voluntarily provided fecal pellets collected after 3 pm were homogenized with a hand-held device (Xenox, Fähren, Germany) in 0.9% sodium chloride and diluted appropriately. Then, 1 mL of bacterial suspension was spread on selective plates (3M Deutschland GmbH, Heidelberg, Germany) and incubated at 37 °C overnight. Bacterial growth was investigated one day before, 1 week after and 6 weeks after FMT (see scheme in Figure S1a). In addition, feces of antibiotics-treated mothers was investigated in comparison to untreated dams (Supplementary Figure S2a) as well as transgenic animals that did not undergo pre- and postnatal antibiotics treatment and donor animals (Supplementary Figure S2b). CFUs were counted from whole plates (Enterobacteriaceae) or representative sectors of plates (Lactobacillaceae) and normalized to fecal material weight.

### 2.4. qPCR for Quantitation of Selected Bacteria

Fecal samples were analyzed at the MVZ Institut fuer Mikrooekologie GmbH (Herborn, Germany). Microbial DNA was extracted from 200 mg feces by automated isolation, pipetting, and analysis as described before [9]. Primers were selected to recognize either the whole bacterial phyla (Firmicutes, Bacteroidetes) or main representatives within the murine microbiota (e.g., *Akkermansia muciniphila*, the genus *Bifidobacterium*).

Standard curves were produced, using the appropriate reference organism to quantify qPCR values into number of bacteria per gram (wet weight). Values obtained were normalized to mean of total numbers of sequences obtained.

### 2.5. Behavioral Tests

Behavioral tests were performed within the last 1.5 weeks before sacrifice with a fixed time schedule (see Supplementary Figure S1). Cage labels did not include information regarding the FMT treatment to minimize bias during the experimental procedure. Animals were tested according to their cage numbers; thereby receivers of old and young donor-derived cecal material were tested alternatingly.

#### 2.5.1. Nesting Test

Nest building is a proxy for general animal wellbeing but also for cognitive integrity [40,41]. Mice were habituated to paper stripe material (Ssniff Spezialdiäten, Soest, Germany) as described previously [42], receiving 10 g of material per cage. On the scoring day, the quality of the nests was scored in the morning latest at 9:00 a.m. and non-integrated material weighed. As nest building also depends on body temperature, this was measured by an infrared thermometer (Braun, Lausanne, Switzerland) in the anal-genital region.

#### 2.5.2. T-Maze

The T-maze was conducted using equipment as described in [43]. Briefly, animals were habituated for 3 min in the starting arm, followed by two days of subsequent testing (4 runs each day). Arm entry was assessed by Anymaze (version 6.1; Stoelting Europe, Dublin, Ireland) and a video camera system (Imaging Source, Bremen, Germany).

#### 2.5.3. Neophobia Test

Mice were positioned in a 10 × 10 cm box with closed exit within an open field arena enlighten by a strong spotlight (halogen floodlight, RITOS type 6095115 AIP44, 150W, Ritter Leuchten GmbH, Mömbris, Germany). Exit door was removed after 2 min and the time measured up to when the mouse was outside the box with all four paws.

#### 2.5.4. Radial Arm Water Maze

The radial arm water maze was performed as described previously with slight modification [44]. In brief, seven trials per mouse were conducted with every second run having a hidden platform up to the fifth run; afterwards platform was hidden in run 6 and 7. Entry errors and swimming time were recorded by Anymaze software (version 6.1; Stoelting Europe, Dublin, Ireland) and the videocamera system (ELP, Shenzen, Guangdong, China). If a mouse did not reach the platform within the one-minute test duration, it was guided by hand to the platform. Animals were able to expose themselves to a red warming light after the test runs.

Locomotor function of animals was checked to exclude a potential direct impact of antibiotics on mobility that might compromise the observed behavior (for example swim speed was measured as 0.09998 ± 0.03656 m/s for wild type (wt); 0.09793 ± 0.01585 for young group; 0.1045 ± 0.02838 for old group; *p* > 0.83 for all comparisons).

### 2.6. Sacrifice and Tissue Preparation

Six weeks after FMT, animals were anesthetized by isoflurane inhalation and sacrificed by decapitation. Truncal blood was collected and serum gained after a minimal clotting time of 45 min by two-times centrifugation (1680× *g*, 10 min, 10 °C and 15,680× *g*, 10 min, 10 °C). Duodenum and one brain hemisphere were drop-fixed in 4% PFA. The second brain hemisphere was snap-frozen in liquid nitrogen and stored at −80 °C until further use.

### 2.7. LBP ELISA

Serum was applied to LBP ELISA as recommended by the vendor (Origene, Rockville, MD, USA). 10 µL of serum were used per well.

### 2.8. Histology

Duodenal cross-sections were stained with standard hematoxylin and eosin (HE) staining procedure. Briefly, samples were de-paraffinized in xylene, re-hydrated with a series of ethanol dilutions, and, then, stained for 5 min in hematoxylin solution. After washing for 10 min in tap water, counter staining with eosin took place for 40 s, followed by dehydration (ascending ethanol concentrations), xylene treatment and embedding with Entellan (Merck Chemicals GmbH, Darmstadt, Germany).

Parameters such as villus length were measured by using ImageJ (Rasband, W.S, MD, USA) after blinding of the samples towards the experimenter. Four measurements of each parameter (villus length, crypt depth, muscular layer, and submucosa thickness) were conducted for every slice by using a clock-wise orientation to avoid selection bias (structures were measured at 3, 6, 9, and 12 h when seeing the gut cross-section as a clock).

Aβ from heterologous overexpression was detected in different brain regions as described previously [32] using the antibody 6E10 (RRID:AB_662804, Covance, Princeton, NJ, USA). Two slices per mouse were stained and anonymized by a person not involved in the investigation. Staining intensity was assessed by using Aida image analyzer 4.26 software (Raytest, Straubenhardt, Germany) as described before [32].

ThT stain was conducted following Ly et al. [45] using 40 µm slices and 1% ThT (Sigma Aldrich, Steinheim, Germany) in ethanol. Images were acquired using the Zoe Fluorescent Cell Imager (Bio-Rad, Feldkirchen, Germany) microscope and analyzed by ImageJ software (Rasband, W.S., MD, USA). Unspecific background staining was subtracted.

### 2.9. Western Blotting

Prefrontal cortex (PFC) specimens were dissected on chilled metal plates and homogenized in 10 mM Tris/HCl pH8 supplemented with protease inhibitor (Roche, Basel, Switzerland) using a Tissue Lyzer and chilled steel beads (Qiagen, Hilden, Germany). Protein concentration was measured with a BSA standard curve and Roti Nanoquant (Roth, Karlsruhe, Germany). Then, 17.5 µg of protein were subjected to a 12% PAA gel and transferred to nitrocellulose by tank blot method. Primary antibodies were as follows: 6E10 for detection of human APP (Covance, Princeton, NJ, USA), anti-NOS1 (RRID: AB_626757, SantaCruz, Dallas, TX, USA), anti-GFAP (RRID:AB_2631098, Cell Signaling Technology, Danvers, MA, USA), anti-GAPDH (RRID:AB_561053, Cell Signaling Technology, Danvers, MA, USA), and anti-BDNF (RRID:AB_2039756, Alomone, Jerusalem, Israel). Appropriate secondary antibodies labelled with HRP were used together with the SuperSignal West Femto chemiluminescent substrate (Thermo Fisher Scientific, Waltham, MA, USA) and a CCD camera (Raytest, Straubenhardt, Germany). Signals were densitometrically analyzed as described for histological sections.

### 2.10. Statistical Analyses

Statistical significance was determined by Student’s t-test with additional Welsh-correction if needed. For results with included wild type control, one way ANOVA was performed with Fisher’s LSD post-hoc test (Graph Pad Prism 6 and 8). Normal distribution was tested with Anderson–Darling or Shapiro–Wilk test. A *p*-value of <0.05 was assigned statistically significant. Animals were assigned to treatment groups by dedicating treatments alternatingly to ascending ear marking numbers before the genotyping (transgenic animal with lowest ear marking number = young, …). Data are presented as mean + SEM.

## 3. Results

### 3.1. FMT Procedure and Proof of Efficacy

**Figure 1:**
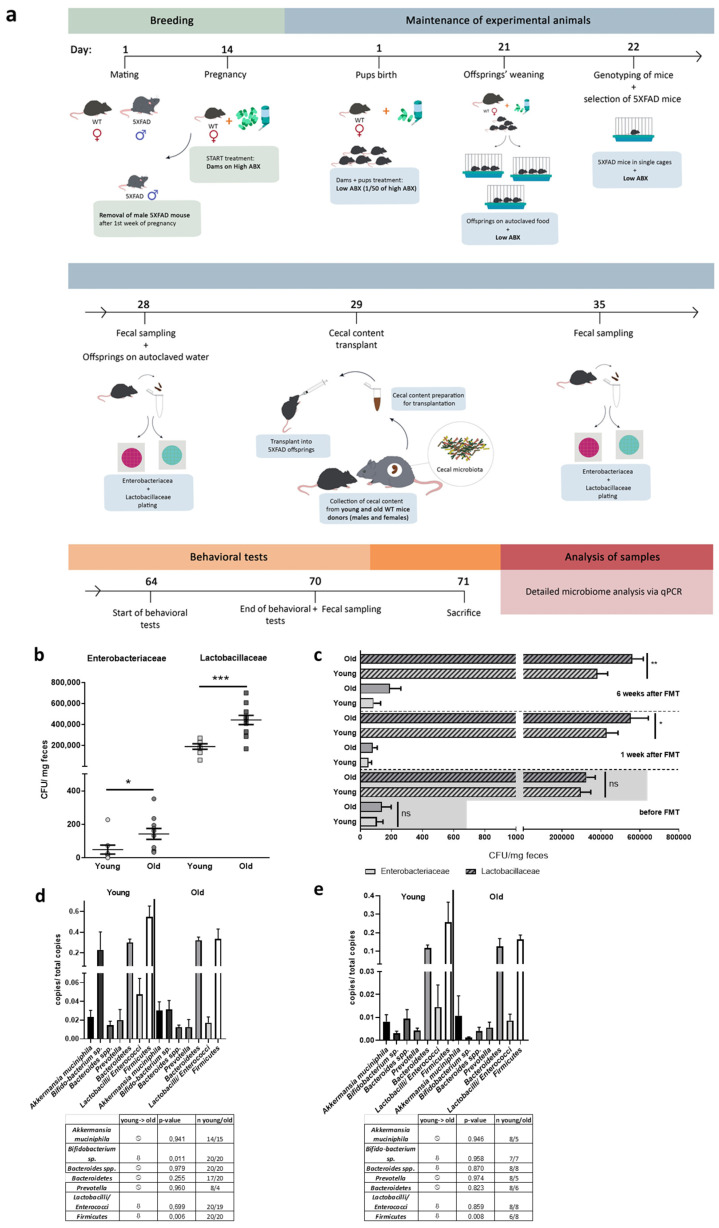
FMT after pre- and postnatal antibiotics treatment of 5xFAD model mice. (**a**) Dams were subjected to antibiotics (ABX) treatment via drinking water after two weeks of pregnancy (high ABX). With giving birth, antibiotics dosage was reduced (low ABX) and offspring was kept on this dosage until one day before FMT at the age of about four weeks. Fecal sampling was performed directly before, one week after and six weeks after FMT. (**b**) Wild type mice aged four weeks (young) or one year (old) were used as donors for FMT. CFUs/mg of feces for Enterobacteriaceae and Lactobacillaceae were assessed by using selective culture plates (*n* = 8 for young; *n* = 10 for old). (**c**) CFUs of animals subjected to FMT were counted on selective culture plates during the experiment (*n* = 20 per group, 10 female). 5xFAD mice treated with cecal material from young wt mice are designated as “young” while animals treated with material from one year old wt mice are designated as “old”. Samples from transgenic mice without any treatment served as control to monitor the efficacy of the antibiotics’ treatment regime (values given by the height of the grey boxes, see Supplementary Figure S2b). (*, *p* < 0.05; **, *p* < 0.01; ***, *p* < 0.001). (**d**) Besides cultivatable exemplary bacteria, several groups of bacteria were analyzed by qPCR using fecal gDNA from animals six weeks after FMT and specific primer pairs (“young”: 5xFAD treated with cecal material from young wt mice; “old”: 5xFAD treated with cecal material from old wt mice). The table beneath the graph summarizes findings, analyzed independent samples, and *p*-values (one way Anova with Fisher’s LSD test). (**e**) For a parallel analysis of the microbiome of untreated young and 1 year old wild type mice, the same analysis as described in (**d**) was performed on fecal material-derived gDNA of mice without antibiotics-treatment or FMT.

To investigate the impact of FMT donor age, we treated 5xFAD mice, after partial depletion of the gut microbiome by antibiotics administration, with diluted cecal material following the scheme in Figure 1a. Donors were aged 4 weeks (designated as young) and thereby age-matched to experimental animals or 1 year old (designated as old). Firstly, we analyzed whether possible differences in the selected microbiota specimens occur between young and old donors. We differentiated microbiomes by mere CFU counting of specific families: Enterobacteriaceae were nearly threefold and Lactobacillaceae were twofold increased when comparing old versus young donor group (*p* = 0.048 for Enterobacteriacea; *p* = 0.0003 for Lactobacillaceae; Figure 1b).

Efficacy of the antibiotics’ treatment was proven by quantifying CFU from dams included in the experiment in comparison to CFUs obtained from untreated dams (samples were collected one day after weaning to preserve pups from possible causative stress during sample collection). For Enterobacteriaceae a reduction by factor 43 was found in antibiotics treated dams (*p* = 0.046, Supplementary Figure S2a), while Lactobacillaceae decreased by factor 62 (*p* = 0.0012, Supplementary Figure S2a). The effect of the treatment was weaker when analyzing four weeks old offspring directly before FMT but still significant (reduction in comparison to untreated mice: factor 6 for Enterobacteriaceae, *p* = 0.049; factor 2 for Lactobacillaceae, *p* = 0.002; Supplementary Figure S2b). The groups assigned to the treatment arms were indistinguishable before FMT (*p* = 0.9996 for Enterobacteriaceae and *p* = 0.6594 for Lactobacillaceae, Figure 1c). One week after FMT, both bacterial family counts in animals having received fecal material from young age-matched donors were indistinguishable from mock-treated transgenic mice (see Supplementary Figure S3). Lactobacillaceae were significantly increased in the group receiving material from old donors in comparison to animals receiving FMT from young donors, while Enterobacteriaceae counts tended to be higher (86 versus 40 CFU/mg) but without reaching statistical significance. After six weeks the difference for Lactobacillaceae was still observed; Enterobacteriaceae were increased from 44 CFU to 200 CFU but *p*-value remained below significance level (*p* = 0.0664).

For a further estimation on the efficacy of FMT, gDNA of fecal material derived from animals six weeks after FMT was subjected to qPCR-based analysis. For assessing the influence of age on the selected bacterial groups, untreated wild type mice with the same age (young = 4 weeks; old = one year) were investigated. Due to the fact, that these animals did not receive an antibiotics treatment, a direct comparison of bacterial counts between both paired groups shown in Figure 1d ande is not feasible. *Akkermansia muciniphila*, *Bacteroides* spp. as well as *Lactobacilli*/*Enterococci* ratio remained unaffected by age. However, the decrease in the Firmicutes was robust and significant in both, 5xFAD mice receiving cecal content from old wild type donors and aged wild type mice without further treatment as compared to the two respective control groups. A decrease in Bifidobacteria could be observed in 5xFAD mice having received cecal material from aged mice (0.229 counts versus 0.031 counts/ total counts). A similar trend occurred in the untreated aged wild type mice, but did not obtain statistically significant results (0.003 counts versus 0.001 counts/total counts).

### 3.2. Physiological and Behavioral Changes Evoked in 5xFAD Mice by FMT Donor Age

**Figure 2:**
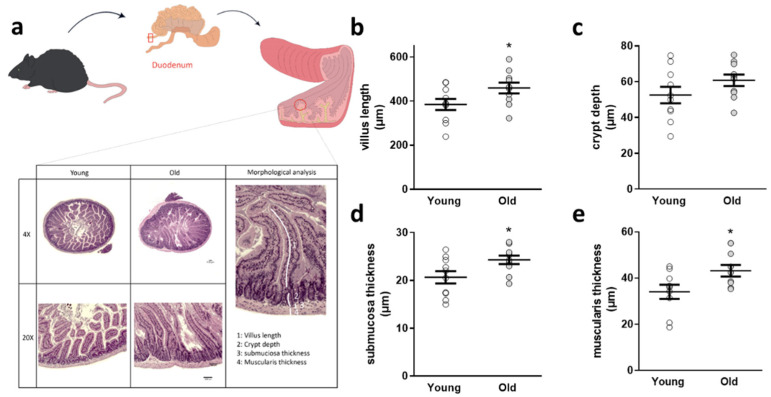
Duodenal architectural parameters assessed in 5xFAD mice after FMT. (**a**) Duodenal sections were stained with HE and villus length (**b**), crypt depth (**c**), submucosal thickness (**d**), and muscularis thickness (**e**) measured. Exemplary sections are shown for each group. Two slides per animal (*n* = 10 animals per group; 5 female) were used and four villi and neighboring structures taken into account per slide. (*, *p* < 0.05). Data are presented as mean ± SEM. Single data points present the mean of all technical values derived from one animal (“young”: 5xFAD treated with cecal material from young wt mice; “old”: 5xFAD treated with cecal material from old wt mice).

Because the gut is the first organ system to monitor effects of an altered intestinal microbial community, we analyzed duodenal architecture subsequently (Figure 2a): while crypt depth was not changed, villus length, submuscosal thickness, and muscularis thickness were significantly increased in 5xFAD mice receiving cecal material from old donors (Figure 2b–e) six weeks after FMT.

**Figure 3:**
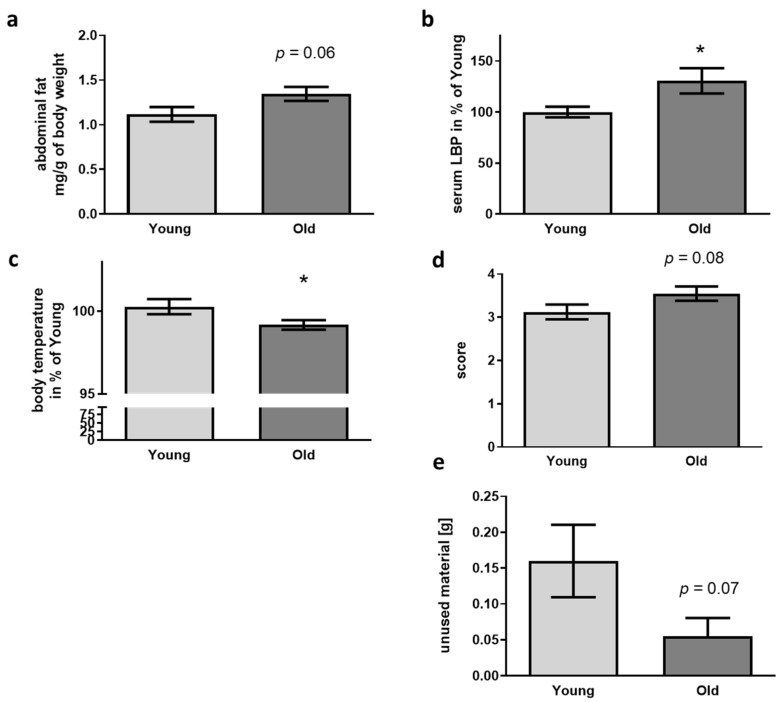
Physiological parameters and nest building capability in 5xFAD mice after FMT. (**a**) Abdominal fat was isolated and weighed after dissection (*n* = 11 per group; “young”: 5xFAD treated with cecal material from young wt mice; “old”: 5xFAD treated with cecal material from old wt mice). (**b**) LBP was quantified from serum samples (*n* = 11 per group). (**c**) Body temperature was assessed directly before sacrifice using an infrared thermometer (*n* = 8 for young and 10 for old; temperature was only taken into account if the animals were not defecating or urinating during measurement procedure). (**d**) Nest quality was scored, and material not integrated in the nest weighed (**e**) (*n* = 20 per group, 10 female). Data are presented as mean ± SEM (*, *p* < 0.05).

Aging is also accompanied by increased size of body fat pads in mice [46,47]. When quantifying the amount of abdominal fat per g of body weight, no significant elevation but a trend for increased fat amount occurred in recipients of old cecal material (Figure 3a). Breakdown of the gut-blood barrier and leakage of LPS from the gut into the blood stream is another epiphenomenon occurring in aged mice (e.g., [48]). In this regard, we found a significant increase of about 25% of LPS-binding protein (LBP, Figure 3b) when comparing mice treated with cecal material from old with those treated with material from young donors.

Not only in humans but also in mice, aging can be accompanied by a drop in body temperature: mice after FMT with old donor material showed a reduction of about 1% as compared to animals treated with material from young donors (Figure 3c). Huddling and nest building are two strategies for behavioral temperature regulation in mice [49]. According with this, nest building quality was—even if not obtaining statistically significant—elevated in the receivers of old donor cecal material and amount of unused material decreased (Figure 3d,e).

**Figure 4:**
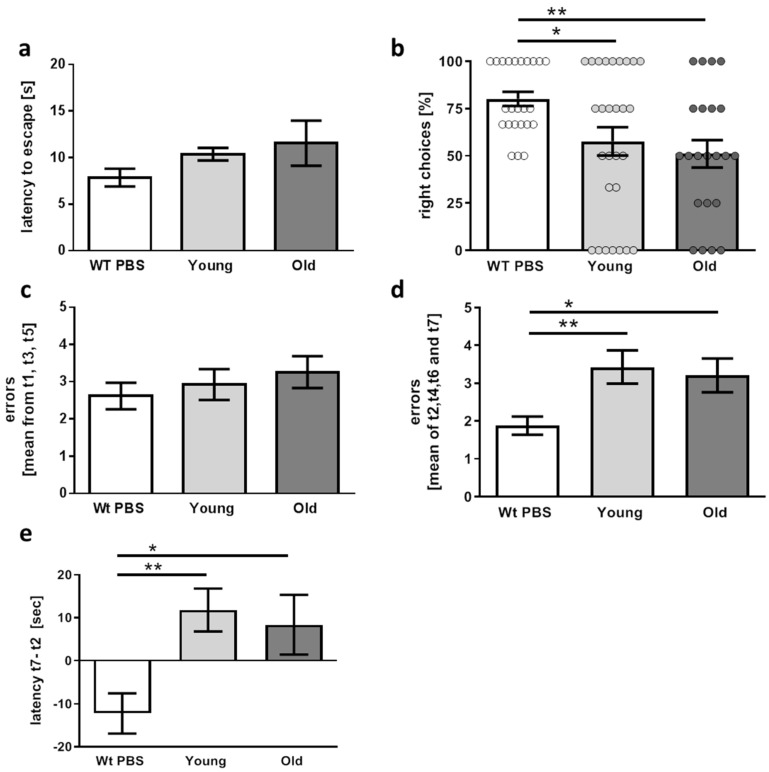
Performance of 5xFAD mice in behavioral tasks six weeks after FMT. (**a**) Latency to enter a brightly lit arena was measured as an anxiety-reporting parameter (*n* = 13 for wild type and 11 for transgenic mice treated with cecal material from young or old donors (designated “young” and “old”)). (**b**) Percentage of right choices during two different testing days in the T-maze were assessed. Dots represent values measured from the individual days. (**c**) The radial arm water maze was applied with alternating visible and hidden platform up to trial 5, the last two trials were performed with hidden platform only. To exclude visual deficits, errors made with visible platform were counted within three trials (t1, t3, and t5). (**d**) Learning and short-term memory were tested within the trials with a hidden platform (t2, t4, t6, and t7). (**e**) Difference of latency for finding the hidden platform between trial 7 and trial 2 was calculated. (**b**–**e**) *n* = 13 for wild type, *n* = 14 for transgenic mice treated with cecal material from young and 11 for mice treated with old donor material. Data are presented as mean ± SEM (*, *p* < 0.05; **, *p* < 0.01).

Our hypothesis was that if a microbiome affected by aging of the host is causative to AD pathogenesis, this FMT should result in aggravated “symptoms”. AD-like pathology manifests in 5xFAD and other transgenic mouse models with learning and memory deficits and in some cases also with altered anxiety. Therefore, mice were subjected to different behavioral tasks. To prove applicability of the respective test system, wild type littermates from litters not treated with antibiotics were included in these tests. First, neophobia was tested by measuring latency to enter a brightly lit arena (Figure 4a). Wild type mice escaped fast with a mean latency of 7.8 s. Mean values were higher in transgenic mice (10.4 and 11.5 s) but differences did not reach significance—neither in comparison to the wild type animals nor by comparison of “young” and “old” group (wt versus young *p* = 0.238; wt versus old *p* = 0.087).

For spatial orientation in the T-maze test, superiority of the wild type animals was also found: while 75% of these mice selected the appropriate alternating arm in the maze, only about 50% right choices were made by the transgenic AD model mice, independently of FMT treatment (Figure 4b). Between the treated groups, no significant change was detected (*p* = 0.4778).

Learning and orientation was also tested in a radial arm water maze, a task for fast assessment of AD-related deficits such as working memory (ability to avoid arms already used for escaping during each trial) and reference memory (ability to avoid arms without escape platforms). All three groups of mice did not differ in regard to errors made while the platform was visible (Figure 4c). This indicates that general visual ability was not compromised. In addition, swimming speed was indifferent (see methods description). However, finding the hidden platform resulted in more errors in the transgenic mice in comparison to the wild type controls (Figure 4d) and, also, in a longer difference in latency time to find the platform as measured between the first trial with the hidden escape platform and the last trial (Figure 4e). However, 5xFAD mice did not present significant differences in both investigated parameters (errors as well as latency difference) after FMT from old donors as compared with animals treated with cecal material from young donors (*p* = 0.6883 and *p* = 0.6656).

### 3.3. Aβ Deposition and Disease-Related Markers in the Brains of FMT Receiving 5xFAD Mice

**Figure 5:**
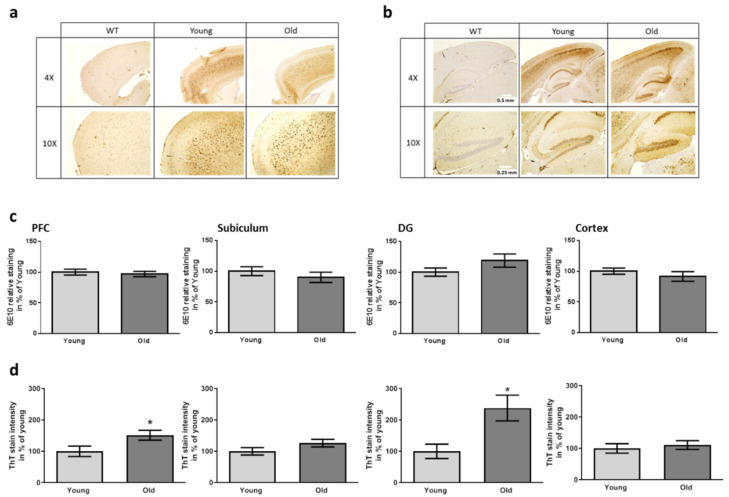
Aβ and plaque staining in brain regions of 5xFAD mice after having received FMT. The area of the prefrontal cortex ((**a**), PFC) and the hippocampal area ((**b**), DG: dentate gyrus) were stained with antibody 6E10. Scale bars represent 0.5 and 0.25 mm (4× and 10× magnification). Exemplary pictures in comparison to a wild type sample are shown. (**c**) Quantitative analysis of the staining intensity using defined area-sizes (as described previously [32]). (**d**) Quantitation of ThT-dependent plaque staining. For exemplary pictures of this staining please see Supplementary Figure S4. Two slides per mouse were analyzed. Data are presented as mean ± SEM in % of the mean of animals treated with caecum content from young donors ((**c**,**d**): *n* = 9 for transgenic mice treated with cecal material from young and 10 for mice treated with old donor material (designated “young” and “old”) (*, *p* < 0.05)).

In sum, none of the applied behavioral tests was able to discriminate between the two receiver groups of the FMT while AD model mice were mostly clearly to distinguish from wild type controls. Nevertheless, age of the 5xFAD mice was in general comparably young with 10 weeks and symptoms were probably just about to rise. Molecular changes, however, in humans have been described to occur even decades before clinical symptoms become observable (e.g., [50]). Therefore, we analyzed deposition of Aβ-peptide containing material in the brain of recipient animals. The mere amount of Aβ-dependent staining detected with the human-specific antibody was comparable for both recipient groups of FMT for all investigated brain areas (Figure 5a–c). Additionally, plaques were stained with Thioflavin T. Here, a higher plaque load was observed in mice receiving FMT from old donors in the prefrontal cortex and the dentate gyrus as compared to the group with young FMT donors (Figure 5d; Supplementary Figure S4 for an exemplary picture). This allows the assumption that while expression of the transgene or its processing to Aβ is not altered, deposition is enhanced.

**Figure 6:**
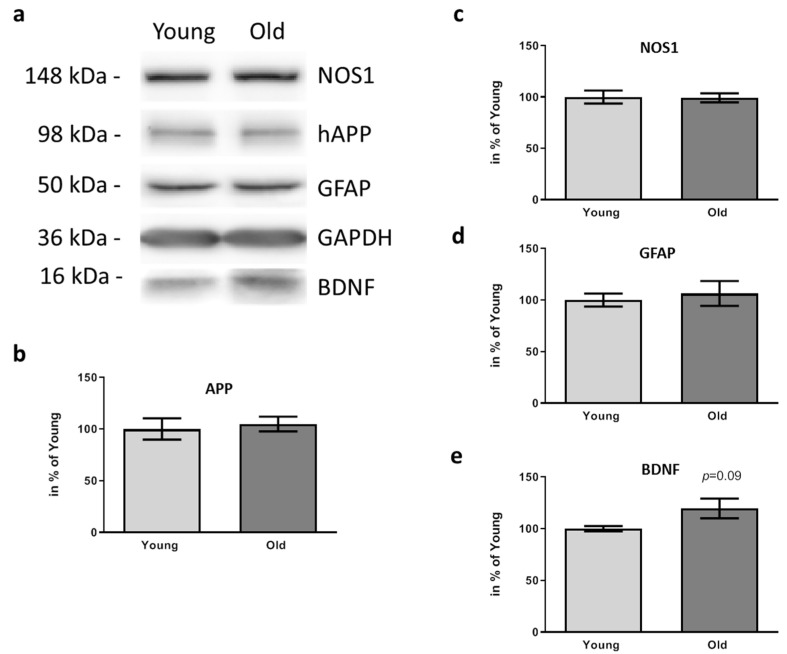
Effects of FMT donor age on inflammation and BDNF amount within PFC of 5xFAD mice. The PFC area of 5xFAD six weeks after treatment with cecal content form young or old wt mice was dissected and 17.5 µg of protein subjected to Western blotting after homogenization. Mice receiving material from young donors are designated “young”, those receiving material from old donors are designated as “old”. A representative sample pair for each of the detected proteins is shown (**a**). GAPDH served as a normalization control for all other detected signals. Quantitation (**b**–**e**) was performed based on sample sizes of *n* = 6–7 per group and values are presented as mean ± SEM in % of the mean of animals treated with caecum content from young donors (“Young”).

Due to the finding that plaque deposition in PFC and DG was affected by the FMT from old donors, we next analyzed key molecules with an already identified correlation to AD pathogenesis in PFC. Firstly, we quantified levels of human APP and confirmed again that the elevated plaque amount did not simply rely on increased protein amount (Figure 6a,b; *p* = 0.7167).

Next, we analyzed NOS1 and GFAP as molecules reporting on inflammatory status: AD model mice having received FMT from young or old donors revealed the same protein levels of both proteins (Figure 6a,c,d). BDNF, in contrast, (Figure 6e) showed a slight increase, which did not reach statistical significance level.

## 4. Discussion

Taking into consideration the immense amount and diversity of commensal microorganisms and their metabolites, and acknowledging their function besides the pathologic representatives, has added a certain degree of dynamism to the AD research field where a certain fatigue seems to have occurred due to the lack of effective evaluation of clinically applied drugs. Nevertheless, knowledge on the altered “AD microbiome” is restricted and trials on FMT for treatment of neurological disorders have to be seen as pioneer work. Here we explored the age of the donor, providing material for FMT, to see whether this might have an impact on disease hallmarks in a prominently used AD rodent model—the 5xFAD mouse. Following a previously published antibiotics treatment regime [33], we were able to reduce the amount of bacteria within the murine gut which provided a platform for inoculation with a FMT-derived community. There is intense debate regarding the use of antibiotics or germ-free mice as a basis for such investigations [51,52]. Germ-free mice from gnotobiotic facilities might be affected by developmental defects such as decreased barrier function of the gut (e.g., [53]), or altered behavior such as anxiety due to impact on the HPA axis [54]. Antibiotics seem—in contrast to establishing a mouse line in a germ-free condition—to represent the easier approach. This method, however, provides its own pitfalls as impressively demonstrated for AD mouse models: already in 2016, the anti-amyloidogenic effect of a microbiome-depletion approach was published [33,55]. Early post-natal antibiotics treatment (P14–P21) resulted not only in long-term alterations of gut microbial genera but also in reduction of brain Aβ deposits and neuroinflammation in aged APPSWE/PS1ΔE9 mice. Several publications revealed that antibiotics such as vancomycin, bacitracin, and metronidazole might affect metabolic homeostasis—especially brain insulin sensitivity—in different mouse strains [56,57,58,59]. This might be highly relevant for the observed effects in AD mouse models as a diabetic phenotype contributes to pathogenesis and C57Bl6 mice, often used as disease models, are prodiabetic—in particular, males are prone to obesity. Nevertheless, we here applied antibiosis as the method of choice to empty the ecological niche of the gut before FMT as we only intended to compare the two groups of transgenic mice that would be similarly affected by the treatment. In addition, a recent study showed that antibiotics used within our study seem not to be able to penetrate into the brain [60]. However, some behavioral domains might be affected by dam and early-life antibiotic treatment such as anxiety [61,62], therefore we cannot exclude that in the comparison to the untreated wild type littermates, the antibiotics-treated 5xFAD mice of both groups (young and old) were inferior due to the drug treatment in addition to their genotypic defects.

As described in the original work reporting on the here used parameters of FMT [35], the transferred microbiota seemed to be stable after six weeks as assessed by CFU counting for Enterobacteriaceae and Lactobacillaceae. These two families represent important examples of the microbiota community and have been shown to be affected by aging [63]. On one hand, Enterobacteriaceae include mostly pathobionts, pathogenic bacterial species presented in small concentrations in a physiologic healthy gut, which can expand under certain conditions (e.g., inflammation), provoking infections when the host defense mechanisms fail as a result of the aging process [64,65]. On the other hand, Lactobacillaceae have been associated with health functions such as protection of the host against pathogens, improving the intestinal barrier function and promoting metabolic and immunologic pathways in the gut [66]. By this, they can be assumed to act as anti-aging commensals [67,68]. Therefore, both families were used as a proxy for assessing microbiota status concerning viable cells. Additionally, we analyzed microbiota composition via qPCR-based analysis of selected bacterial groups. The main finding was that Firmicutes were reduced both in 5xFAD mice after having been treated with cecal content from old animals and untreated aged, 1 year old wild type mice in comparison to their respective controls while Bacteroidetes were unaffected. A diminished Firmicutes to Bacteroidetes ratio has been correlated with age in humans before with a ratio of 10.9 in adults and 0.6 in the elderly [69]. For mice, data are inconsistent, maybe also due to differences in strains, investigated age, and housing conditions. However, a recent publication reported that the highest amount of Firmicutes was found in 6-month-old mice with a subsequent decline in aged individuals [70]. The decrease in *Bifidobacteria*, which has been described to accompany aging [71,72,73], only got statistically significant for the animals receiving FMT from old donors. However, our control group of untreated young and aged wild type mice was comparably small; therefore, we cannot exclude that this explains the lack of a comparable statistically significant finding in this sub-group of mice.

Besides the change in fecal microbiota composition, FMT also resulted in altered duodenal architecture as for example observed for villus length. Such an elevation of villus length in aged animals has been observed before when comparing 2–3-month-old and aged mice (20–22 months, [74]) and is probably related to decreased intestinal stem cell regenerative potential. Additionally, the increased thickness of the submucosa and the muscularis has also been found in NMRI/Bom mice within duodenum when comparing 12-month-old animals with young animals [75]. Therefore, we assume that the FMT did not only affect the microbial community but also subsequently the organ system in direct contact to the microbiota and its metabolic products. Abdominal white fat pads are also known to increase with age (e.g., [46]). Here, we could only demonstrate a tendency to increase in mice receiving cecal material from old donors, which did not reach statistical significance. Probably, this can be attributed to the fact that our investigation stopped already six weeks after FMT. However, the possibility that FMT did not only directly affect the gut, but also had systemic consequences became obvious with quantifying LBP in serum. Interestingly, LBP was not only associated with age-dependent elevated intestinal permeability in humans and rodent models but also with increased abdominal fat [76,77]. In accordance with these signs of FMT-driven pro-aging effect, a lowered body temperature was observed in 5xFAD mice having received FMT from old donors. Loss of thermoregulation as a sign of aging has been described in mammals in general [78]. However, data on mice are highly inconsistent which might be due, for example, to different amount of handling stress when animals are restrained for temperature assessment. Nevertheless, a study using sensors inserted under the loose skin of the pelt of mice was able to show a drop in body temperature with aging [79].

In previous publications, we used nest building capability as a proxy for hippocampal function in AD model mice aged 12 weeks or older [8,32]. Nest architecture is based on hippocampal function as has been demonstrated by lesion models and by identifying so-called nest cells within this brain area [40,80,81]. In this regard, it has to be taken into consideration that nest building is an important factor of thermoregulation and depends on ambient and probably also body temperature [82]. In our study, animals were still too young to expect defects in nest-building due to AD pathology; however, animals with FMT from old donors displayed the tendency to build better quality nests and to integrate more material which might be explained by their reduced body temperature.

As animals in our study were still comparably young with a final age of 10 weeks, severe behavioral deficits were not to be expected in general. Mostly, behavioral deficits have been described in 5xFAD mice with an age of at least 4–6 months, depending on the respective test or even sex of the investigated groups [8,83]. In the neophobia test, no changes in anxiety-dependent behavior, i.e., longer latency to leave the box, could be detected. In the T-Maze test for spatial orientation, already at this young age deficits for 5xFAD mice as compared to wild type littermates became obvious and, also, in the radial arm water maze. Considering the mentioned behavioral tests, the latter allows a fast evaluation of mice learning and orientation taking advantage of natural motivation provided by immersion in the water and by scoring errors [44]. Instead of the mainly used Y-maze, we used the T-maze, which forced the animals for a more distinct choice. Probably by this, we were able to unravel very early behavioral symptoms of the 5xFAD mice. Nonetheless, no differences occurred between animals with old donor or young donor FMT.

Although analysis of cortical slides revealed that the general amount of Aβ-containing material within the brain was not elevated by the treatment with old donor cecal material, increased plaque deposition in two central regions for AD pathogenesis—PFC and hippocampus DG [84,85]—was found. This elevated plaque deposition was not based on increased Thy1-dependent expression of the human APP as shown by protein quantitation in PFC. Increased gut permeability (leaky gut) has been observed in the elderly and may facilitate the infiltration of immune cells in the brain and thereby accelerate the neuroinflammation in AD [86]. However, elevated signs of inflammation were not observed as demonstrated by measuring NOS1 and GFAP protein amount. Injection of LPS—intraperitoneally or directly into the brain—has been shown to elicit microglia activation in rodents and to activate NO production [87,88]. Together with our finding that serum LBP increased in the animals subjected to FMT from old donors, this is somehow contra-intuitive. The peripheral LBP increase was only limited (ca. 25%) and we did not check for increased LBP or LPS within brain parenchyma. That means that presumably no additional LPS entered the brain while it was present in the periphery. Interestingly, BDNF was increased in the “old” group. Per se, this molecule has been described to decrease within the aging brain in humans and rodents [89,90] and more manifested decrease was found in *postmortem* AD brains or liquid biopsies [91,92,93]. In very early stages of AD, however, an increase has been reported [94] and we assume that the cecal material from the old donor mice here started a vicious development—just observed at its emergence.

In sum, we here provide evidence that a single-time FMT is able to transfer certain geronto-physiological aspects in mice and that six weeks after FMT selective hallmarks of AD were aggravated by microbiota from an aged donor. If such a treatment would also be sufficient to evoke further behavioral changes besides the increased body temperature and corresponding improved nest building, has to be investigated in future with a more prolonged post-inoculation phase or with repeated FMT.

## Data Availability

Data is contained within the article or Supplementary Materials.

## Supplementary Materials

The following are available online at https://www.mdpi.com/article/10.3390/microorganisms9122548/s1, Figure S1: Schedule for behavioral testing, Figure S2: Antibiotics treatment of mothers and offspring, Figure S3: Comparison of CFU from 5xFAD with and without age-matched FMT, Figure S4: Prefrontal cortex slice as an example for ThT staining. Table S1: Litter characteristics. Litters from antibiotic-treated and control dams giving birth in the same period were compared. While sex ratio is indifferent, the size of the litters from antibiotics-treated dams (ABX) was even elevated (unpaired *t*-test, * *p* = 0.027).
