# Supplementary material for: Impact of the Age of Cecal Material Transfer Donors on Alzheimer’s Disease Pathology in 5xFAD Mice
Source: Microorganisms. 2021 Dec 9;9(12):2548. doi: 10.3390/microorganisms9122548 (PMC8708188; doi:10.3390/microorganisms9122548)
Supplement: Supplementary file 1 [file microorganisms-09-02548-s001.zip › microorganisms-1446985-supplementary.pdf]

	Littersize		n	sex ratio	
	**mean**	**SD**		**mean**	**SD**
**control**	6,10	2,34	21	1,38	1,31
**ABX**	7,57*	1,78	21	1,58	1,00

Supplementary Table S1: Litter characteristics
Litters from antibiotic-treated and control dams giving birth in the same period were compared. While sex ratio is indifferent, the size of the litters from antibiotics-treated dams (ABX) was even elevated (unpaired t-test, *p=0.027).

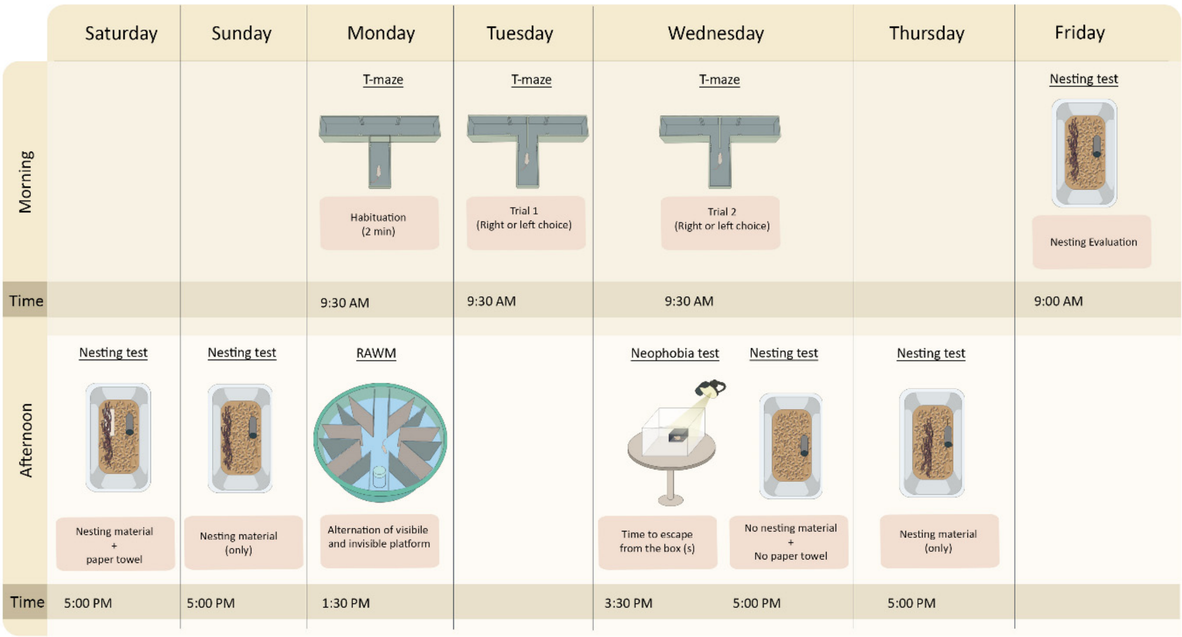

Supplementary Figure S1: Schedule for behavioral testing

Mice were tested within one week before sacrifice as indicated in Figure 1. Starting times for each test are indicated in the scheme.

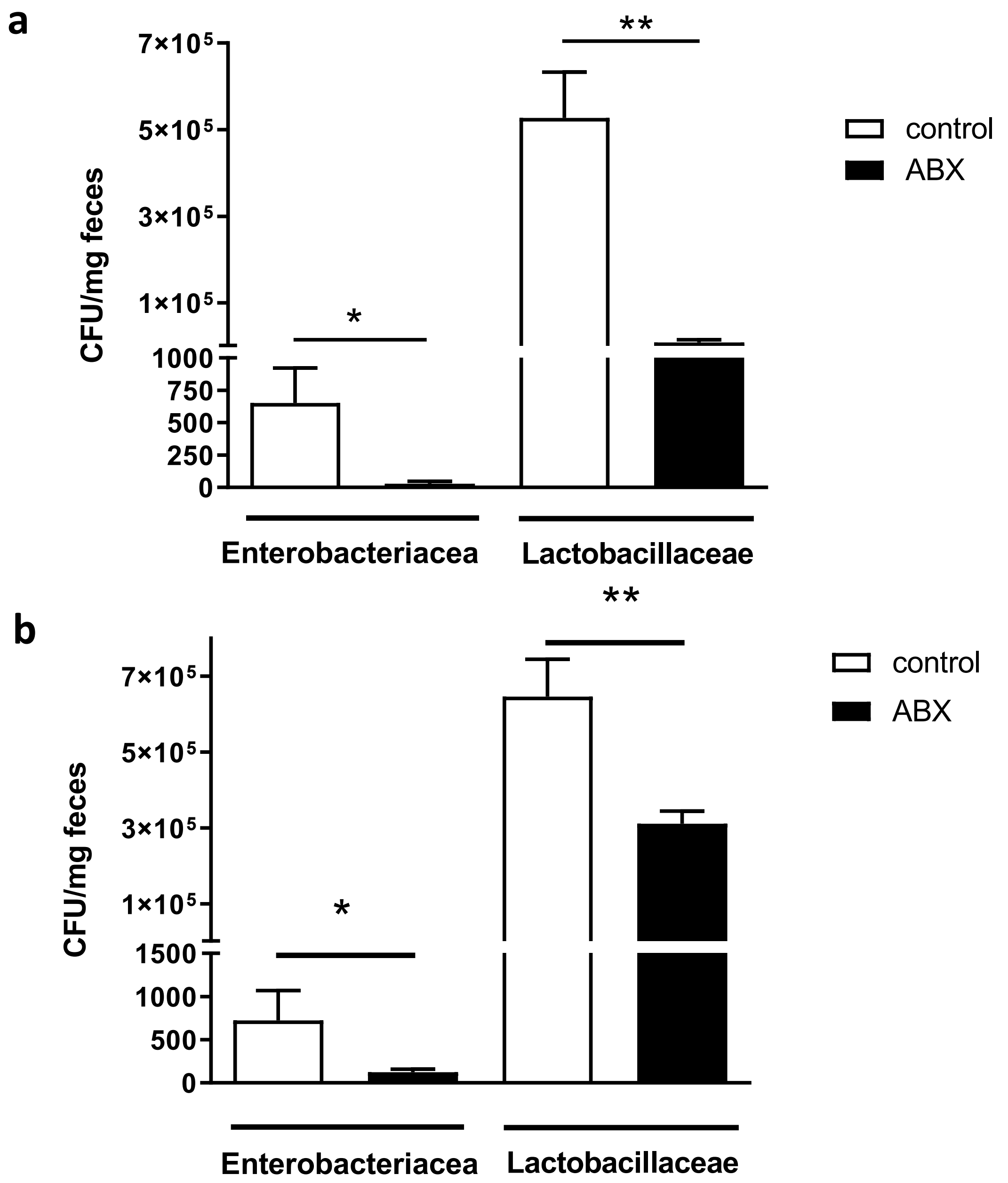

Supplementary Figure S2: Antibiotics treatment of mothers and offspring

Suspended and diluted fecal material from dams (a, n=9 per group) or offspring (aged 4 weeks) was subjected to specific bacterial growth plates and colony forming units (CFU) counted after 24 h of incubation. Animals were either treated with antibiotics (ABX, n=38) or with normal drinking water without supplements (control, n=18).

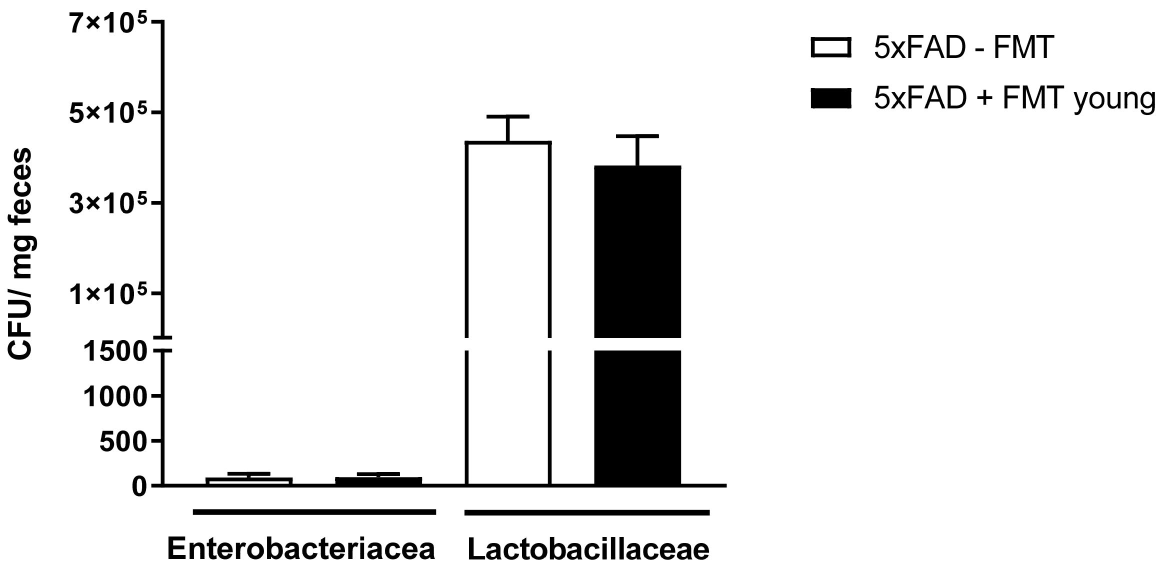

Supplementary Figure S3: Comparison of CFU from 5xFAD with and without age-matched FMT
Suspended and diluted fecal material was analyzed as described and colony forming units (CFU) counted after 24 h of incubation. Animals were either treated with PBS (-FMT, n=15) or with cecal material from age-matched (young donors) (+FMT young, n=20). Data are presented as mean + SEM.

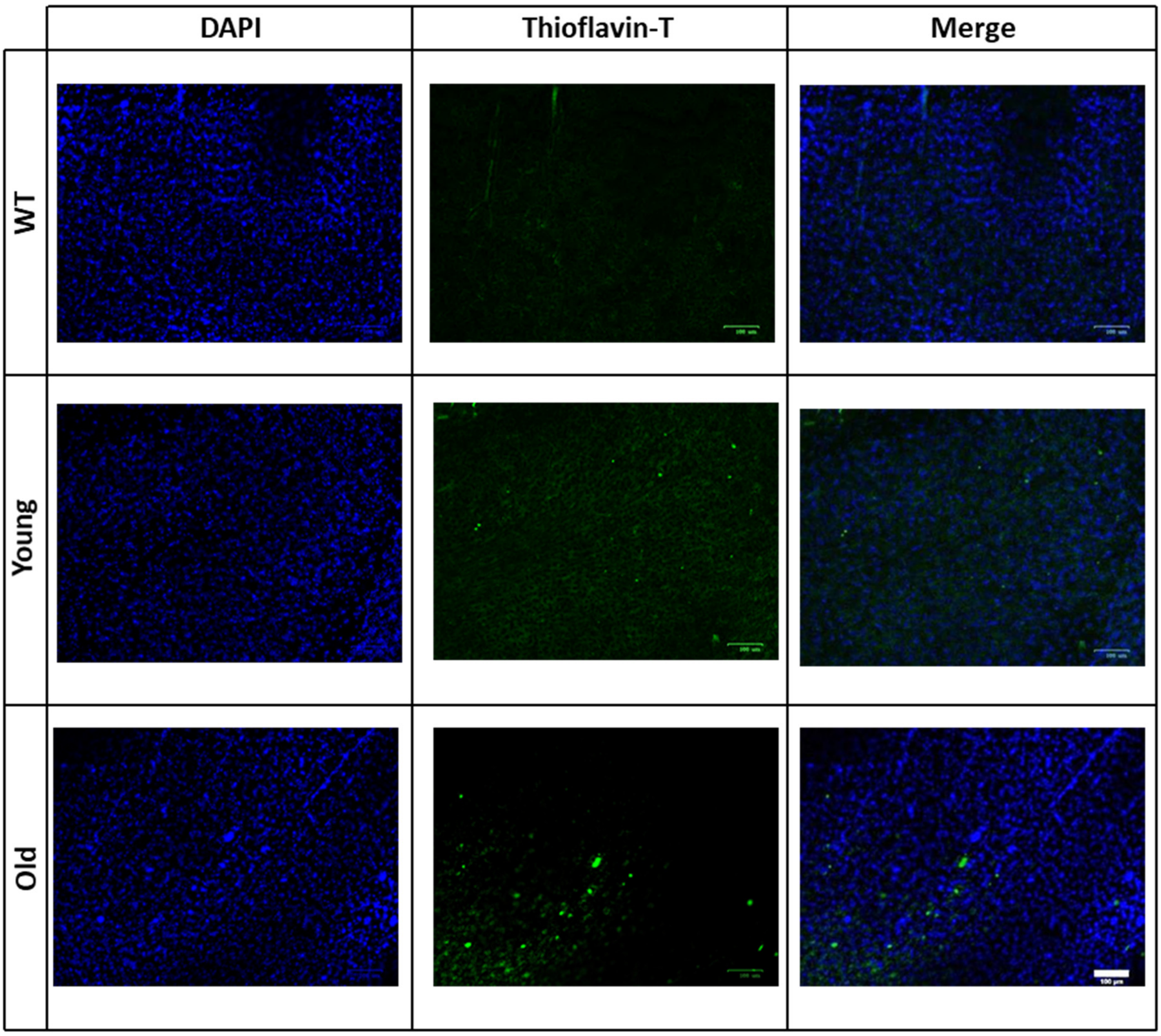

Supplementary Figure S4: Prefrontal cortex slice as an example for Thioflavin-T staining

Slices from prefrontal cortex (PFC) from wild type (wt) animals or 5xFAD mice receiving fecal material from young (designated “young”) or old donors (designated “old”) were stained with Thioflavin-T (ThT) for Aβ aggregates and DAPI for neuronal nuclei labeling. Exemplary pictures and the merge of both signals are shown. Scale bar: 100 µm.
